# Falls and Fall-Prevention in Older Persons: Geriatrics Meets Spaceflight!

**DOI:** 10.3389/fphys.2017.00603

**Published:** 2017-10-11

**Authors:** Nandu Goswami

**Affiliations:** ^1^Gravitational Physiology, Aging and Medicine Research Unit, Institute of Physiology, Medical University of Graz, Graz, Austria; ^2^Department of Health Sciences, Alma Mater Europea University, Maribor, Slovenia

**Keywords:** immobilization, orthostatic intolerance, countermeasures, spaceflight, aging, bedrest, fraility, falls

## Abstract

This paper provides a general overview of key physiological consequences of microgravity experienced during spaceflight and of important parallels and connections to the physiology of aging. Microgravity during spaceflight influences cardiovascular function, cerebral autoregulation, musculoskeletal, and sensorimotor system performance. A great deal of research has been carried out to understand these influences and to provide countermeasures to reduce the observed negative consequences of microgravity on physiological function. Such research can inform and be informed by research related to physiological changes and the deterioration of physiological function due to aging. For example, head-down bedrest is used as a model to study effects of spaceflight deconditioning due to reduced gravity. As hospitalized older persons spend up to 80% of their time in bed, the deconditioning effects of bedrest confinement on physiological functions and parallels with spaceflight deconditioning can be exploited to understand and combat both variations of deconditioning. Deconditioning due to bed confinement in older persons can contribute to a downward spiral of increasing frailty, orthostatic intolerance, falls, and fall-related injury. As astronauts in space spend substantial amounts of time carrying out exercise training to counteract the microgravity-induced deconditioning and to counteract orthostatic intolerance on return to Earth, it is logical to suggest some of these interventions for bed-confined older persons. Synthesizing knowledge regarding deconditioning due to reduced gravitational stress in space and deconditioning during bed confinement allows for a more comprehensive approach that can incorporate aspects such as (mal-) nutrition, muscle strength and function, cardiovascular (de-) conditioning, and cardio-postural interactions. The impact of such integration can provide new insights and lead to methods of value for both space medicine and geriatrics (Geriatrics meets spaceflight!). In particular, such integration can lead to procedures that address the morbidity and the mortality associated with bedrest immobilization and in the rising health care costs associated with an aging population demographic.

## Introduction

This paper outlines a general overview and comparison of spaceflight medicine, the experience of human aging, as well as important connections and parallels between spaceflight physiology and the aging process. Consideration of these connections and parallels can be exploited to improve human health for both astronauts living in microgravity and for older persons on earth. Included here are the following aspects: (1) Gravity and its effects on physiological systems; (2) Spaceflight induced physiological effects; (3) Aging induced physiological effects; (4) Earth-based simulation of spaceflight deconditioning compared with the experience of bedrest immobilization in older persons; (5) Maintenance of astronaut health: current countermeasures against the negative effects of microgravity in space; and (6) Benefits of life in space and how it can help life on Earth: Geriatrics meets spaceflight! These aspects are now outlined in the sections below.

## Gravity and its effects on physiological systems

During the course of natural evolution, as species moved from water to land, the effects of gravity needed to be countered. Evolving land animals, especially those that changed posture from a horizontal to vertical orientation, including humans, needed to develop new systems to regulate fluid distribution and blood flow, to ensure structural support as well as to maintain postural stability and facilitate locomotion (see Morey-Holton, 2003).

The extent to which gravity influences physiology is often underestimated. Gravity affects many systems and structures such as, size and location of internal organs (e.g., heart). Lillywhite (1988) investigated these factors in detail. He reported that snakes that live in trees, on land or in the sea have different locations of the heart, which in turn, affects their gravity tolerance (as assessed by loss of consciousness with increasing gravitation loading as provided by centrifugation). For instance, during centrifugation, the greatest gravity tolerance was seen in tree snakes (in whom the heart is closest to the brain) while the least tolerance was seen in water snakes (in whom the heart is located in the central part of the body and far away from the brain; Lillywhite et al., 1997).

In humans, physiological systems have evolved in such a way as to compensate for the effects of gravity. For example, even though the heart is located below the brain during standing, the force of contraction of the heart and heart rate are adequate to maintain blood flow to the brain. Furthermore, during standing, the pooling of blood in the legs due to the gravitational force directed earthward is countered by the muscle pump in the lower limbs, by one-way venous valves as well as via the “respiratory pump.” Physiological systems in healthy humans are well adapted to gravitational changes induced by standing (orthostasis) and thus changes in posture do not normally lead to any significant problem. This ability to stand without physiological problems is termed orthostatic tolerance.

## Spaceflight induced physiological effects

The powerful influences of gravity (or the lack of it) on physiological systems can be seen when gravity is reduced (e.g., the microgravity environment of spaceflight). Spaceflight produces observable changes in the physiology of humans, many of which are almost impossible to control during missions, and all of them lead to time-dependent adaptation processes (Blaber et al., 2013; Hargens et al., 2013). These adaptive changes show inter-individual differences (Goswami et al., 2013). Entering microgravity leads to the alteration of physiological processes, that depend significantly on gravity, and introduces physiological functional changes such as, adaptations in cardiovascular control functions, cerebral perfusion (Blaber et al., 2013; Goswami et al., 2013), and changes in the musculoskeletal system and a reduction in sensorimotor system performance.

Antonutto and di Prampero (2003) reported that cardiovascular deconditioning in spaceflight is a significant problem, which is related to the time spent in microgravity. The homeostatic adjustments in the cardiovascular system in spaceflight include alterations in autonomic regulation, decrease in blood pressure and decreases in blood, plasma, and interstitial fluid volumes (Gazenko et al., 1986). The shift of fluid toward the head is followed by a reflex-induced decrease in total blood volume (Levy and Talbot, 1983). Over a few hours, a new equilibrium is established for blood returning to the heart with decreased stroke volume and diastolic blood pressure (Antonutto and di Prampero, 2003).

Returning to normal gravity on Earth has many important effects, including increased heart rate, decreased vasoconstriction (increased venous pooling), a reduction in exercise capacity (as quantified by heart rate responses and oxygen consumption during specified work loads, Hamilton, 2008), and decreased orthostatic tolerance (Buckey et al., 1996). Orthostatic intolerance involves exaggerated increase of heart rate, fatigue, light-headedness, dizziness, impairment of performing physical and mental tasks, and often leads to syncope during upright posture. In particular, such orthostatic intolerance is a potential threat to crew safety when re-entering a gravitational field in general. The impact of decreased orthostatic tolerance is not trivial, in that ~30% of astronauts experience orthostatic intolerance following even a short duration spaceflight (Buckey et al., 1996). This number grows to 80% after long duration flights (Meck et al., 2004). The reduction of orthostatic tolerance is an extremely important aspect of cardiovascular deconditioning following actual space flight (Blaber et al., 2011), and can also be observed in several important clinical problems in Earth based medicine. Orthostatic intolerance seems to be common in both American (Bungo and Johnson, 1983) and Russian crew members (Gazenko et al., 1981) returning from space. Reduced tolerance to lower body negative pressure (LBNP) has also been observed (Blomqvist, 1983).

Spaceflight leads to reductions in heart function and changes in heart rate responses, lowering of the total blood volume, and even changes in venous compliance in different vascular beds due to blood pooling. All these factors, either individually or in combination, may be responsible for the decreased venous return that occurs on standing (Watenpaugh and Hargens, 1996) thus leading to post-spaceflight orthostatic intolerance (Blaber et al., 2011). Furthermore, it has been suggested that autonomic neural control alterations, including those of the baroreflex may induce most of the responses seen in the cardiovascular system post-spaceflight (Mano, 2005).

## Aging induced physiological effects

Important parallels can be found between deconditioning due to spaceflight and deconditioning and other physiological changes due to aging. For instance, in humans living on Earth and especially in older persons, hypovolemia and reduced cerebral blood flow, cerebral or peripheral vascular disease, metabolic or endocrine disorders, autonomic neuropathy, or cardiac arrhythmias may result in syncope (dizziness and loss of consciousness) when standing up. For older persons such reduced tolerance to upright posture (orthostatic intolerance) is a common condition that can lead to falling down and injury.

Frail older adults often have decreases in their functional and physiological reserves, as well as malnutrition, which particularly makes them vulnerable to diseases and falls (detailed in Martínez-Velilla et al., 2015). Furthermore, the negative consequences of aging such as, illness or injury due to falls often require admission to hospital. However, the immobilization that occurs during hospitalization is itself a major factor in physiological deconditioning and functional decline and can contribute to a downhill spiral of increasing frailty, orthostatic intolerance and increased risk and incidence of falls (Mühlberg and Sieber, 2004). A systematic review by Heinrich et al. (2010) indicates that fully 0.85–1.5% of all health care costs are dedicated only to falls and their consequences. Furthermore, given that older patients spent up to 83% of hospital admission lying in bed (Lazarus et al., 1991; Brown et al., 2009; Pedersen et al., 2013), confinement in bed during hospitalization—and its effects, for example, orthostatic intolerance —represents a central challenge in the care of the vulnerable older population in any acute care hospital across the whole world.

## Earth-based simulation of spaceflight deconditioning compared with bedrest confinement in older persons

Bedrest has been used routinely as an analog for studying the consequences of weightlessness on body systems as seen during space flight (Pavy-Le Traon et al., 2007; Jost, 2008; Goswami et al., 2015a). Sandler and Vernikos reviewed the first 25 years of bedrest studies in 1986 and proposed inactivity as a tool to study space flight effects (Sandler and Vernikos, 1986).

The bedrest study protocol, in which subjects lie in supine position over variable time periods, is a highly controllable experimental set-up which provides excellent possibilities to investigate physiological function changes during lowered gravitational stress (Trappe et al., 2007; Arzeno et al., 2013; Cvirn et al., 2015; Florian et al., 2015; O'Shea et al., 2015). Bedrest is also an important platform that allows for the testing of (potential) countermeasures to overcome the physiological deconditioning effects of lowered gravitational loading due to microgravity as well as the effects of bedrest deconditioning during hospitalization (Schneider et al., 2009; Waha et al., 2015). Insights and countermeasures to combat deconditioning obtained from these experiments would also be of great value in addressing the problem of increasing frailty in older persons which could arise due to bedrest confinement.

## Maintenance of astronaut health: current countermeasures against the negative effects of microgravity

For maintenance of their health in space—and to counter difficulties of re-entry into Earth's atmosphere as well as post-flight orthostatic intolerance—astronauts carry out exercise regimes comprised of resistive exercise and physical training of up to 2.5 h per day for 6–7 days per week (Hackney et al., 2015; Petersen et al., 2016). Petersen et al. (2016) recently reported that since the advanced resistive exercise device (ARED)—which provides an opportunity to increase the prescription of resistive training—was introduced at the international space station (ISS) 8 years ago, the resistance exercise component of total in-flight exercise has increased but cycle ergometry and treadmill running has decreased. The usage of ARED is particularly important since cycle ergometry performed in space does not seem to increase workload despite the need to achieve this in microgravity. This could be due to technical and biomechanical factors associated with exercising in space, which provides additional physiological and technical constraints. Similarly, when carrying out treadmill running exercise in space, crewmembers exercise with static loads of only between 70 and 80% of their bodyweight (detailed in Petersen et al., 2016). Overall, the current devices for exercise available on the ISS and the physical activity training regime appear to better address some of the issues related to astronaut health.

Just like physical activity, *nutrition* is important for maintenance of human health on Earth and in space. Aspects such as inadequate nutrition intake, vitamin D status, and oxidative damage need to be considered in human spaceflight (Smith et al., 2005). However, only up to 70% of energy intake requirement is typically met in space crew members (Smith et al., 2009, 2013). Unsurprisingly, inadequate food/energy intake in space leads to body weight loss and could affect, muscular, cardiovascular, and endocrine systems (Smith et al., 2009). For instance, caloric restriction in obese humans following weight reduction therapy, in fasted pilots and from animal experiments have shown that even caloric restriction in moderate amounts can affect fluid homeostasis, as well as lead to decreases in blood volume, heart rate, blood pressure, and norepinephrine and cardiovascular function (Florian et al., 2015). In addition, caloric/fat restriction has been shown to lead to alterations in baroreflex function, altered vascular smooth muscle tone, and consequently, decreased orthostatic tolerance (Florian et al., 2015).

Recent missions, however, have shown that crew energy intake can be maintained at > 90% of the required amount (Smith et al., 2012). Nutrition alone, however, has not been shown to be effective in preventing lower limb muscle volume or strength loss. Adequate *energy intake* + *resistance exercise training* during long-term spaceflight has been shown to improve lean muscle tissue, reduce muscle volume loss and muscle strength loss (Trappe et al., 2007) and to reduce fat (Smith et al., 2012). Furthermore, nutrition and exercise in space have been shown to help in counteracting bone loss (Smith et al., 2012): Bone resorption in space was accompanied by bone formation.

Potential nutritional countermeasures that may help in bone protection during spaceflight include (Smith et al., 2013):

- Dietary *omega-3 fatty acids* or eicosapentaenoic acids: These fatty acids with virtually no side effects have been shown in cellular, ground analog, and spaceflight environments to counteract weightlessness-induced bone loss by inhibiting NF-kappaB activation (nuclear factor ‘kappa-light-chain-enhancer’ of B-cells; Zwart et al., 2010).
- *Vitamin D supplementation*: As spacecraft are particularly shielded from the harsh ultraviolet light exposure, and there is a lack of *vitamin D*, supplementation of this vitamin is particularly important for space crewmembers. Therefore, vitamin D (800 IU/day) is supplemented in spaceflight (Smith et al., 2012). To what extent, however, maintenance of vitamin D in space prevents bone loss needs to be further explored.
- *Vitamin K supplementation*: The role of this vitamin in preventing bone loss in space is not clear: some crew have shown lowering of vitamin K status but recent data from spaceflight and bedrest show that vitamin K status does not change (Zwart et al., 2011).

As we embark on long-term exploration of deep space, nutrient intake must be optimized. Therefore, research aimed at optimizing/supplementing the effects of physical exercises via additional nutritional supplementations and/or pharmacological interventions is continuing (detailed in Smith et al., 2012; Hackney et al., 2015). Additionally based on data from ground based (bedrest) studies, dietary countermeasures such as *decreasing sodium intake* (currently >5 g Sodium/ day is ingested, Smith et al., 2012) and *how protein intake* affects bone health are also currently being investigated in space.

## Life in space and how it can help life on earth: geriatrics meets spaceflight!

From the above discussion, it can be seen that many key problems of aging and age-related deconditioning have parallels with those problems confronted during spaceflight and thus synergies can be drawn between aging and spaceflight. For instance, older persons spend up to 80% of their time in hospital bed-confined, which, in turn, effects their physiological functions in a manner similar to spaceflight deconditioning. The strategies used in space for maintenance of astronaut health could also be used for the benefit of life on Earth, especially in older persons (Geriatrics meets spaceflight!). For instance, just as in space, caloric restriction on Earth has been shown to slow the aging process, increase stress resistance and delay the onset of age-related diseases, and even provide cardioprotection (Han and Ren, 2010).

In the following section, aspects related to bedrest confinement induced physiological deconditioning as well as the need to provide rapid interventions (countermeasures) to alleviate the deconditioning are discussed, as well as how current countermeasures used in space could be potentially used in geriatrics.

### Aging, immobilization, and early remobilization

Ambulation is a prerequisite for mobility, and mobility is a primary requirement for autonomy and quality of life. However, toward the end of an individual's lifespan, reduced muscle function due to, for example, sarcopenia and dynapenia impair ambulatory function. The addition of injuries or illnesses renders people often unable to ambulate, and, therefore, muscles become deprived of the habitual stimulating signals. Combination of sarcopenia and disuse atrophy causes profound muscle wasting (Kortebein et al., 2008; Suetta et al., 2009), and the deconditioning process is a medical risk in itself that causes numerous adverse events (Brown et al., 2004). These consequences, related to reduced ambulation and increased bed confinement, begin immediately after admission to hospital, and deficits in the Activities of Daily Living (ADL) can be seen even on the second day of bedrest confinement in older people (Hirsch et al., 1990). If the deconditioning passes below the threshold of independent ambulation, then the deficits in muscular strength and power become perpetuated. Such muscle deficits are difficult to recover from in older patients, as most of them have generalized inflammation and metabolic disorders.

**Figure 1:**
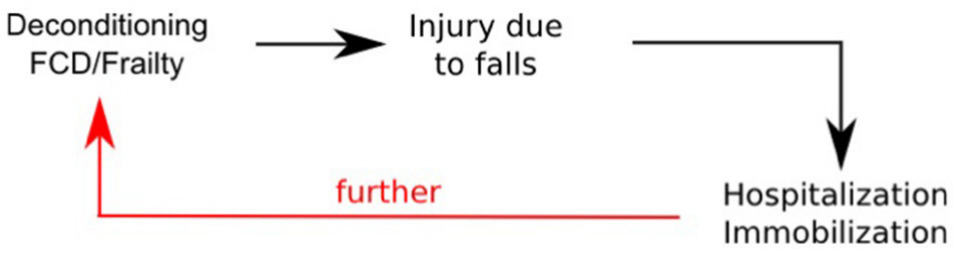
The vicious cycle of frailty, immobilization, and falls. FCD: Functional and cognitive decline.

To effectively address this challenge, it is necessary to identify and intervene in pathways that link age-associated frailty, undernutrition and other factors to the effects of immobilization as for example in the cycle depicted in Figure 1.

During bedrest confinement, immediate action is therefore required to break the vicious circle of immobilization and muscle wasting: effective remobilization has to start as early as possible during and after hospital admission. Intervening in this cycle requires a holistic multifactorial approach that takes into account key factors such as, (mal-) nutrition, (de-) conditioning, muscle loss, cardiovascular, and vestibular effects; all of these could contribute toward immobilization induced orthostatic intolerance. It is important to note that since astronauts returning to Earth from the microgravity environment of spaceflight show similar physiological deconditioning as bed-confined persons, effective management of these effects is important for both astronauts and older persons.

### Addressing both ambulation and falls in older persons

Falls in older people are particularly frequent during hospital stays or in the weeks and months thereafter (Mahoney, 1998). While compromised muscle function undoubtedly contributes to falls in old age, there are many other reasons for the increasing number of falls in older people (Blain et al., 2016; Bousquet et al., 2017). One of the key factors responsible for falls in older persons is postural hypotension (Weiss et al., 2004). Furthermore, it is well known that bed rest confinement induces hypovolemia (Convertino, 2007) and orthostatic intolerance (Dittmer and Teasell, 1993), the latter being perceived as dizziness upon standing up. Of note, 40% of falls in nursing homes are related to posture changes from supine to standing (Rapp et al., 2012). Also, anti-hypertensive and diuretic medicine treatment is a known cause of falls in older people (Gangavati et al., 2011). It is very likely, therefore, that the excessive risk of falls after hospital discharge is attributable to compromised cardio-postural control and impaired cerebral perfusion. Therefore, there is a need to study both falls in ambulatory persons and falls following bedrest confinement, and assess the inter-relationship between cardio-postural control and the risk of falls.

For maintenance of standing balance, the ability of the body to detect postural disturbances and respond accordingly is required. These abilities deteriorate with increasing age, thus predisposing older people to imbalance and greater risk of falls. Aging is also associated with worsening of the somatosensory and motor systems functions, which in turn, can lead to poor static standing balance. A research model to measure and assess the cardiovascular-postural system as an integrated and interacting system, which can be used to determine the effectiveness of both the postural and cardiovascular systems (both of which are severely impacted by immobilization, particularly in older persons) has been developed (Blaber et al., 2009; Goswami et al., 2012). Current knowledge proposes the measurement and tracking of changes in the two systems and their interaction in order to detect and assess changes due to immobilization that can impact cardiovascular, postural, or interactive processes in older persons. This assessment strategy, carried out via a sit to stand test followed by gait monitoring, will allow for the delivery of specific therapies to improve ambulatory recovery and also provide feedback on the effectiveness of physical interventions during immobilization.

As most astronauts also experience orthostatic intolerance post-spaceflight, there is a need to develop countermeasures that can alleviate orthostatic intolerance in the deconditioned returning astronaut. Astronauts replace spaceflight-induced plasma loss via salt ingestion shortly before landing to prevent post-spaceflight orthostatic intolerance (Campbell and Charles, 2015). Similarly, replenishment of plasma volume loss using salt tablets and fluid loading following 12 days of 6-degree head down bed rest has been shown to prevent post-bed-rest orthostatic hypotension (Waters et al., 2005). However, the impact of plasma volume loss due to bedrest confinement during hospitalization is currently not well-documented and needs to be further explored. It should be proposed that bed-confined older persons should also undergo regular assessment of the plasma electrolytes and—depending on the duration of bedrest confinement (Stuempfle and Drury, 2007)—be provided replacement of the plama volume losses that occur.

### Countering the impact of immobilization during hospitalization in older persons

When older patients are bed confined, a rapid decline in muscle mass, bone mass, and functionality sets in (Singh et al., 2008; Martínez-Velilla et al., 2015). If remobilization starts early, then the declines incurred are small enough to allow recovery (Singh et al., 2008). If intervention is started later, then recovery is usually incomplete and patients can be left with chronic reduced function (Singh et al., 2008; Martínez-Velilla et al., 2015). In many cases unfortunately, remobilization starts too late and patients permanently lose their independence and autonomy; this is associated with higher morbidity and mortality (Singh et al., 2008).

**Figure 2:**
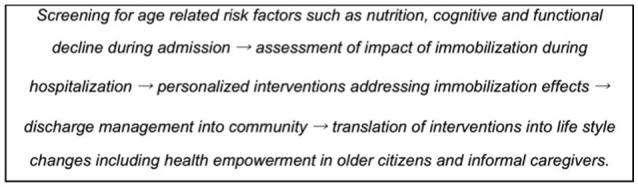
A schema showing the need for patient screening, in-hospital interventions during immobilization and extension of interventions during follow-up in the community.

The interaction of pre-existing factors such as, malnutrition and/or sarcopenia with the effects of immobilization poses complex challenges to managing health in older persons. Furthermore, innovative methods and care procedures implemented during immobilization need to be extended to the period after hospitalization and return to the community to maximize recovery and reduce frailty, to restore mobility and physical activity, and to avoid falls and new hospitalizations. This important dimension has not been investigated yet but knowledge in this regard is urgently needed. An optimal sequence of intervention steps is described in the box below (Figure 2).

Remobilization of older people in the acute care setting is delayed in many hospitals. Physical exercise interventions are often started too late and based upon care provider experience and non-standard decision making, and often started when there is already a substantial muscle mass and function loss. Many older patients pass beyond the “point” of no return for sustainable physical functioning, thereby entering the vicious circle of de-conditioning and hospital re-admissions and further dependency care. Therefore, there is a need to start the interventions as soon as possible (Singh et al., 2008; Martínez-Velilla et al., 2015). Intervention combinations could incorporate physical exercise, nutrition and cognitive-behavioral training. Based on above observations regarding orthostatic intolerance and deconditioning in astronauts, it is reasonable to expect insights from space medicine in regards to these issues.

### Physical activity and exercise training in older persons

In older persons physical activity appears to be beneficial for both physical and mental well being (Olanrewaju et al., 2016). However, there is almost no literature related to the effects of physical activity during bedrest confinement in older persons. While manual physiotherapy is routinely used in bedrest-confined patients, and is highly beneficial in preventing immobilzation-induced contractures and possible thrombotic events, the extent to which this is beneficial in terms of muscle strength and muscle function has not been studied in detail.

Older ambulatory persons often have access to exercise machines, which are often difficult to use in bed-confined patients. Such devices as powerplates and/or vibration plates are routinely used in ambulatory geriatric care. However, currently such devices and interventions are not used in bed-confined patients. Proposing resistive vibration exercises for bed-confined older persons, which have been shown to maintain muscle strength and function in experimental bedrest studies, of varying durations, in young bedrested persons (Schneider et al., 2009) provides an important example of how evidence from ground based analogs of spaceflight could be used in geriatrics. Schneider et al. (2009) reported that carrying out alternating aerobic and resistive exercise during bedrest confinement in young women leads to restoration of the aerobic capacity. This effect, however, is not seen with interventions that only involve nutritional supplementation.

### Role of nutrition: current evidence related to (mal-) nutrition in older persons

There is growing evidence that there is a strong association between risk of frailty and inadequate intake of food in elderly people (Martone et al., 2013). In a large multinational study with more than 4,000 older participants (mean age of 82.3 years: 75.2% female), malnutrition prevalence was 22.8% (Kaiser et al., 2010). Malnutrition prevalence varied in different settings: prevalence in rehabilitation > in hospital > in nursing home > in community. When examining the combined database, the “at risk” group prevalence was up to 46% (Kaiser et al., 2010). In a German malnutrition study prevalence at hospital admission was even higher: between 35 percent (60–70 years) and 60 percent (>70 years) were already malnourished or at risk for malnutrition (Pirlich et al., 2006).

Malnutrition is characterized by unintended weight loss, loss of appetite, loss of muscle mass and bone mass. It results in functional decline, increased morbidity, preterm dependency, more frequent readmission after hospital discharge, early dependency and institutionalization, and finally increased mortality. Together, these factors result in increased costs for healthcare systems. Low dietary intake is one of the most important factors of malnutrition (Saunders et al., 2015). The vast majority of literature shows that sufficient amount of energy and key nutrients such as, protein, vitamin D, and calcium may help to improve muscle function and to maintain muscle and bone mass (European Commission, 2012; Saunders et al., 2015).

### Role of nutritional therapy in older persons

The evidence regarding the beneficial role of nutrition alone is limited (Schneider et al., 2009). A recent Cochrane review reported that nutritional therapy can reduce healthcare costs but overall the evidence from the studies is too heterogeneous and of limited quality for concluding whether malnutrition or its treatment helps in reducing re-admissions (Muscaritoli et al., 2016). Strandberg et al. (1985) reported that nutritional therapy, along with resistance training, improves muscle mass in older persons. A further study showed that high protein diet, provided by lean red meat, at 1.3 g/ kg/day increases lean tissue mass and muscle strength when it is complemented with resistive training in older women (Daly et al., 2014).

### Role of cognitive training in preventing FCD in sedentary older persons

Cognitive interventions have been used as countermeasures to prevent functional decline and even to promote functional outcomes, especially mobility-related improvements in sedentary seniors (Verghese et al., 2010). Indeed, usage of a computerized cognitive training (CCT) protocol, was shown in a recent study to prevent possible bed rest-associated decline in physiological function as well as cognitive changes (Goswami et al., 2015b; Marusic et al., 2016). The total bedrest confinement in the Goswami et al. (2015b) and Marusic et al. (2016) study—lasting up to 2 weeks—was carried out in healthy 55–65 years olds. CCT intervention was effective in improving cognitive function at the end of bedrest as well as improved dual-task walking condition and reduced gait variability (Marusic et al., 2016). The latter functional changes could potentially lead to a reduction in number of falls following prolonged bedrest. Moreover, CCT during bedrest confinement in older persons also prevented decreases in vascular function changes (Goswami et al., 2015b). Since vasculature function is an important contributor to bedrest-induced orthostatic intolerance, and/or cardiovascular diseases, Goswami et al. (2015b) propose that doing CCT in the immobilization state in older persons could lead to a reduction in the bedrest-induced effects on the vasculature.

## Perspectives

Bed rest studies, in addition to being directly relevant for spaceflight and often employed in spaceflight related studies, can be designed and used for investigation of a number of physiological conditions that arise due to aging or are related to a diverse set of situations involving surgery, injury, or chronic debilitating diseases. This example illustrates how a powerful synergy of information can be developed by connecting the physiological responses to microgravity and the consequences of bed-confinement, reflecting the parallels that can be drawn between aging, microgravity, and immobilization (Vernikos and Schneider, 2010).

There is also a need to better understand the pathways that link healthy status to frailty and functional physical and cognitive decline (FCD). This is important as previously independent senior persons can suddenly be hospitalized and become dependent. Thus, there is a need to reduce progression to frailty among the pre-frail and intervene in persons at risk to avoid frailty [European Innovative Partnership Active Healthy Aging (EIP-AHA) report, 2013]. The interventions related to physical activity and life style modifications discussed in this review could be used for developing guidelines and strategies for preventing the negative consequences of bedrest-confinement and for reducing frailty and falls in older persons. Resistive vibration exercises—which have been shown to maintain muscle strength and function in ground based analogs of spaceflight—could be proposed for bed-confined older persons (Schneider et al., 2009). This is an important example of how evidence from ground based analogs of spaceflight could be used in geriatrics. The integration of knowledge of human physiology under conditions of microgravity and changes in physiology due to the aging process allows for each of these physiological conditions to provide new insights into the other and allows for development of new intervention strategies.

## Author contributions

The author confirms being the sole contributor of this work and approved it for publication.

### Conflict of interest statement

The author declares that the research was conducted in the absence of any commercial or financial relationships that could be construed as a potential conflict of interest. The reviewer SM and handling Editor declared their shared affiliation, and the handling Editor states that the process nevertheless met the standards of a fair and objective review.
